# Honey Production and Climate Change: Beekeepers’ Perceptions, Farm Adaptation Strategies, and Information Needs

**DOI:** 10.3390/insects14060493

**Published:** 2023-05-25

**Authors:** Rafael Landaverde, Mary T. Rodriguez, Jean A. Parrella

**Affiliations:** 1 Department of Agricultural Leadership, Education and Communications, Texas A&M University, College Station, TX 77843, USA; jean.parrella@ag.tamu.edu; 2 Department of Agricultural Communication, Education and Leadership, The Ohio State University, Columbus, OH 43210, USA; rodriguez.746@osu.edu

**Keywords:** adaptability, beekeepers, case study, climate change, El Salvador, honey, honey bees, phenomenology

## Abstract

**Simple Summary:**

Most crops grown globally require pollination to produce food, and honey bees are the most important pollinators. Without honey bees, the food supply would decrease and become more expensive. Climate change threatens honey bees by destroying their habitats and food sources. Therefore, beekeepers must implement farm management practices to adapt to climate change. However, in many developing countries, such as El Salvador, beekeepers lack information about climate change adaptation strategies. In this study, researchers interviewed nine Salvadoran beekeepers to understand how their perception of climate change affects their beekeeping production, the adaptation strategies they implement, and their needs for climate change-related information about beekeeping. The climate change-induced challenges beekeepers experienced included food and water scarcity and extreme weather events (e.g., increase in temperature, rain, and winds). As a result, honey bees are dying because they cannot find enough to eat/drink, their hives are damaged, and they are more prone to pests and diseases. To adapt, beekeepers reinforce their beehive boxes, relocate their beehives, and supplement the honey bees’ food. The beekeepers expressed their need for help formulating supplementary honey bee diets and managing pests and diseases. Because they struggled to understand climate change-related information from the internet, they need information and demonstrations from local sources to improve their adaptation strategies and the health and productivity of their honey bees.

**Abstract:**

Because climate change has severely impacted global bee populations by depleting their habitats and food sources, beekeepers must implement management practices to adapt to changing climates. However, beekeepers in El Salvador lack information about necessary climate change adaptation strategies. This study explored Salvadoran beekeepers’ experiences adapting to climate change. The researchers used a phenomenological case study approach and conducted semi-structured interviews with nine Salvadoran beekeepers who were members of The Cooperative Association for Marketing, Production, Savings, and Credit of Beekeepers of Chalatenango (ACCOPIDECHA). The beekeepers perceived water and food scarcity, as well as extreme weather events (e.g., increasing temperature, rain, winds), as the leading climate change-induced challenges to their production. Such challenges have augmented their honey bees’ physiological need for water, limited their movement patterns, decreased apiary safety, and increased the incidence of pests and diseases, all of which have led to honey bee mortality. The beekeepers shared adaptation strategies, including box modification, apiary relocation, and food supplementation. Although most beekeepers accessed climate change information using the internet, they struggled to understand and apply pertinent information unless they received it from trusted ACCOPIDECHA personnel. Salvadoran beekeepers require information and demonstrations to improve their climate change adaptation strategies and implement new ones to address the challenges they experience.

## 1. Introduction

Climate change is driving universal ecological change [1,2], and its variability controls the “distribution, productivity, and many other aspects of species, ecosystems, and landscapes” [3] (p. 1). Climate change has several implications for species’ abundances and essential living activities, such as reproduction and microhabitat use [3,4]. Species will adopt geographic distribution patterns influenced by their environmental tolerance, local food availability, and interactions with other species [3]. Under the current climate crisis, species have limited options to continue their individual and collective everyday activities, reducing their healthy populations and compromising survival [4,5,6]. Therefore, adaptation to existing or new habitats and evolution to tolerate climatic changes are essential for any species to avoid extinction [6,7].

Insects are the dominant species in diversity, abundance, and biomass [7,8]. Along with several other environmental services, the insect class includes some species carrying out pollination for agricultural productivity, continuing biodiversity, and landscape conservation [8,9,10]. According to [7], most insects can contribute to pollination by transferring pollen from one plant to another. However, “the most intense pollination is carried out by flies, butterflies, and moths; some wasps, beetles, and thrips; and, of course, bees” [7] (p. 71). There are approximately 20,000 species of bees within seven families [11]. According to [12], the greatest diversity of bees is found in the northern hemisphere due to bees’ preferences for moderate temperatures. Bees challenge the distribution patterns of most animal and plant species, which have historically shown a preference for warm, tropical weather conditions [13]. However, the above hypotheses remain unproven on a global scale, and are based on a limited number of monitoring and research efforts. Regarding those efforts, [13] commented that “those few efforts that explore the global distribution of bees are descriptive and are based on comparisons between small, well-sampled areas such as Palm Springs and Riverside in California” (p. 451).

Although wild bee populations are declining due to climatic and environmental changes [14,15], managed bee colonies have increased by 45% worldwide in the last 50 years [16]. This abrupt increase is a response to socio-demographic changes in consumer populations, including more people seeking sustainable and healthy food options [16], and the rising global population increasing the demand for food [17,18]. According to [16], although managed honey bee hives are increasing, “that trend, however, does not keep pace with a 300% increase in bee-pollinated crop production in the same time period” [16] (p. 34). Wild and managed bees experience the impacts of climate change in similar ways [15], although managed bees exacerbate the detrimental impact of climate change on the abundance and diversity of wild bees through interference and exploitative competition and displacement [19,20,21]. Conserving wild bees is significantly more challenging due to the limited control that humans have over their livelihoods and movement patterns [15,22,23]. However, by implementing climate-smart management practices, beekeepers and entomologists can make coping with climate change an easier and more realistic process for managed bees [24,25].

Bees currently top the list of endangered animals for many environmental, ecological conservation, and agricultural production organizations [26,27,28]. These concerns stem from research findings that report declining bee populations [7,8,9]. For example, in the United States, the behavior of bee populations indicates that they will become extinct by 2035 if efforts are not made to protect them and their habitats [29,30]. In recent years, extensive information has been collected to demonstrate how climate change affects bees’ survival and limits their ability to provide ecosystem services [31,32]. According to [33], climate change has drastically reduced bees’ habitats and natural food sources. However, there is less evidence and certainty about how climate changes affect bees’ health [15]. This latter information gap has spurred more research and action to investigate how bees respond and adapt to current climate conditions [34]. In addition, researchers are more interested in identifying and understanding the reasons for the changing population density of bee communities. One of the main limitations in obtaining this knowledge is the lack of records and historical data on bee populations, especially in the developing world [35,36].

Beekeeping is not only a honey-producing agricultural sector, but it is also an essential contributor to food and nutrition security, responsible for pollinating up to 30% of global food production [17]. Therefore, beekeeping is vital to the sustainability of world food supplies. In beekeeping, agricultural management practices are being implemented as mechanisms for adapting to climate change [23,24,25]. Exploring the climatic adaptability of beekeeping requires a place-based approach, considering each climatic zone’s geographical and biological characteristics [37]. Many countries and climatic zones still lack information on beekeeping and necessary climate change adaptability strategies [35,36,38,39,40]. The purpose of the study was to explore Salvadoran beekeepers’ experiences adapting to the effects of climate change.

### Climate Change and Beekeeping in El Salvador

With almost seven million inhabitants, El Salvador is both the smallest and most densely populated country in Latin America [41]. In El Salvador, vulnerability to climate change continues to increase due to soil degradation and loss, as well as a reduction in forest cover [42]. According to [43], in the last 30 years, Salvadoran geography has been characterized by extreme weather events, intense periods of drought, and heavy rains that resulted in floods with human and economic losses. Climate change predictions in El Salvador project a temperature increase of 1.4 °C–2 °C and a precipitation reduction of 2–15% by 2050 [42].

Climate change impacts on Salvadoran agriculture are evident in the reduction in productivity and efficiency at each step of the agri-food production and supply chain [44]. By 2070, between 10 and 29% declines are expected in several essential agricultural products in the Salvadoran diet and economy [42]. Agriculture contributes 4.91% of the Salvadoran GDP, and beekeeping has been one of the sectors in constant growth since 1960 [45]. El Salvador is the second-highest region in honey production in Central America after Guatemala [46]. Conventional and organic production of Salvadoran honey continues to grow— according to government reports, El Salvador exported approximately 1000 tons of honey between 2020 and 2021. During this same time period, national news outlets stated that some beekeepers lost up to 50% of their honey production due to climate change, resulting in a 35% loss in national honey production [47]. No official reports of Salvadoran honey production were found to contradict this information.

## 2. Materials and Methods

### 2.1. Study Site

Chalatenango is one of the 14 states in El Salvador. Bordering Honduras, it is the fifth largest state in the country, with a land area of 2017 km^2^ [48]. Until 2020, Chalatenango had the largest number of registered beekeepers nationwide (*n* = 150) [46]. However, it is assumed that many other beekeepers were not recorded in the last national agricultural census [46]. Chalatenango is ranked third in honey production, with 9561 hives distributed in 366 apiaries totaling an average of 26 hives per apiary [46]. Salvadoran beekeepers are generally dedicated exclusively to producing honey, although some beekeepers combine honey production with the production of other agricultural products, such as fruits, vegetables, and poultry [46].

The Cooperative Association for Marketing, Production, Savings, and Credit of Beekeepers of Chalatenango (ACCOPIDECHA for its acronym in Spanish) was founded in 1998 with 60 members [46]. It obtained legal status in 2005. Currently, ACOPIDECHA has 33 members, 60% of whom identify as men and 30% of whom identify as women, with ages ranging from 20–75 years. ACCOPIDECHA facilitates the export of Salvadoran honey to international markets with the goal of obtaining better prices for its associates. In addition, it offers technical assistance in apiary management, risk prevention, and integrated control of pests and diseases [46].

### 2.2. Data Collection and Analysis

Following recommendations for the transferability of the results, a detailed, rich description of each stage of the research process has been provided [49]. Researchers used a phenomenological case study approach to achieve the study’s purpose. Phenomenological case studies enable researchers to draw novel conclusions from day-to-day lived experiences shared by members of a particular group or case [50,51]. Because researchers aimed to explore Salvadorian beekeepers’ experiences dealing with climatic variability in their honey production, particularly the experiences of ACCOPIDECHA members, a phenomenological case study approach was most appropriate.

All 33 ACCOPIDECHA members were recruited to participate in in-depth, semi-structured interviews, and nine agreed to participate. It is common for phenomenological inquiries to involve six to 20 participants [41], but [52] advised researchers to be more concerned with the quality of data provided by participants rather than the quantity of participants. Therefore, because the nine participants accurately reflected the case under study and were interested and committed to sharing their lived experiences adapting to the effects of climate change, they provided sufficient, high-quality data. Only ACCOPIDECHA members were included in the study due to the availability of their contact information. Beekeepers received personal oral and written invitations to participate in the study from the Principal Investigator (PI). For those who agreed to participate, the PI organized a visit to their house to conduct the interview. Each participant received an informed consent document and a one-page research program description. The participants did not receive an incentive for their participation. The interviews lasted an average of 45 min and were audio recorded with participant authorization.

The study answered the following three research questions: (1) How do Salvadoran beekeepers perceive that climate change is affecting their beekeeping production? (2) What farm management practices are Salvadoran beekeepers implementing as a response mechanism to climate change? and (3) What are Salvadoran beekeepers’ needs and preferences for climate change-related information as it relates to beekeeping? The first two research questions were similar to those of [24], who investigated Italian beekeepers’ perceived climate change effects and climate change mitigation strategies. An interview protocol (Supplementary Material File S1), which included 12 guiding questions designed a priori based on the literature, guided the in-depth, semi-structured interviews conducted to answer the three research questions.

The interviews were conducted in Spanish by the PI, who was in charge of transcribing the recordings into text processing software. The PI, who is a native Spanish speaker and a sociolinguistic competent English speaker, translated the data prior to analysis. A non-professional translator is a valid research resource as long as they are sociolinguistically competent in the native language of the study participants or the language to which the data is being translated [53]. Grounded theory methods, including open and axial coding, were used to identify emergent themes [54]. Emergent themes were classified by categories to answer the proposed research questions. Peer debriefing was implemented by two researchers simultaneously coding the data until they reached an agreement regarding data categorization. A peer debriefing process is a form of inter-rater reliability and is essential in qualitative research to achieve trustworthiness [54,55]. Finally, the participants received the research project findings and were able to provide feedback to verify the information.

Researchers increased the data credibility and addressed research bias by comparing and contrasting multiple data sources. Triangulation was implemented using a cross reference approach, which arranges data from multiple sources for comparison. The triangulation involved interview transcriptions, observation memos (including the PI’s on-site observations during the interview), and farmer records, such as public government honey production and commercialization reports [46]. The triangulation showed convergence among data sources.

## 3. Results

### 3.1. How Do Salvadoran Beekeepers Perceive That Climate Change Is Affecting Their Beekeeping Production?

All beekeepers agreed that local water resources had been reduced in the last 10 years mainly due to droughts and low levels of precipitation that are becoming more common. The beekeepers perceived water scarcity as the leading local challenge for any agricultural production. A1 commented, “The water we have access to is scarce… Before this was not a problem, but it is easy to notice that the wells no longer have the same amount of water… And in the rivers and streams the water levels are lower and lower...But that is to be expected because here it does not rain like it used to... Sometimes we have weeks in the middle of winter [raining season] when it does not rain at all... I worry because everything is arid by the end of the summer [dry season]”. A similar downward trend has followed the food availability for honey bees. Salvadoran beekeepers depend on wild flora as a food source, which has been becoming increasingly scarce in the last 10 years. A4 shared, “flowers are also not the same... Before, it was nice to see the fields full of bellflowers. As soon as winter began, you could see the green areas blooming, which was a good sign of food for the honey bees... Right now, it is different; bellflowers and other flowers do not emerge like before... There are green areas where they no longer bloom at all, and there is nothing we can do... That is when honey bees will look for other food sources”.

Local flora density is not the only food-related issue climate change is inflicting for honey bees. According to A5, the nectar levels that honey bees find in local wildflowers (e.g., bluebells [*Campanula patula*]) have been decreasing. They shared, “Honey bees no longer find the same quantity or quality of nectar in the flowers... I assume that the lack of water in the area affects the flowers’ production of nectar... Personally, I have to manage so that the hives have food, but if there are no flowers or crops nearby, it is difficult for the honey production to be good… A couple of years ago, I had a terrible year; I produced very little and not of such good quality... According to what they [buyers] told me, it was because the bees were not well fed”.

Due to the decline in local water and food sources, the beekeepers expressed concern about the increased energy and time invested by honey bees when searching for these two essential inputs, which can damage the hive’s health and honey productivity. A6 said, “Honey bees by themselves scavenge for water and food… With certainty, I can tell you that they will fly for longer periods of time and to greater distances to get water and food, but that means less production time, and in the end, it is not convenient for me… I strive so that my apiaries have food and water... With water, it is a little easier because I carry it and put it in a barrel, and I try to ensure it is always full... But the food supply is more complicated”.

In addition to the additional workload honey bees face in supplying water and food to their hive, climate change places more demands on their physiology and behavioral development. Some beekeepers argued that the local temperature increase augmented honey bees’ physiological needs for water. For example, A6 shared, “With this heat, the poor honey bees have, like any living being, a greater need for water... And then a problematic cycle is created in which the honey bees are living in burning environments and require more water, which is more and more scarce and to get it, it takes more effort... So I end up with sick hives, dead honey bees, and almost no honey... And I still cannot handle this problem”.

Temperature variability is not the only climate change factor directly affecting honey bees. Beekeepers have noticed that extreme rains and winds limit the honey bees’ collection of water and nectar. A4 explained that honey bees have more limited movement patterns due to local climate activity, emphasizing that when there is wind and/or irregular patterns of rain, they cannot fly, as the weather intensity surpasses their strength. Also, regarding the wind and rain, A3 commented, “When it is blowing strong wind, the bees have to invest more energy to fly and continue collecting the supplies the hive needs… Obviously, when it is raining, the bees have less ability to move, and therefore if it affects the production of honey... It is easy to notice that the hives are not in good shape after a couple of days raining”. A4 also shared that, although it has not happened to him, he knows of beekeepers whose apiaries have been damaged by the winds because they knocked the wooden boxes to the ground or because of things falling on the boxes (e.g., trees).

All climate changes directly or indirectly alter the local livelihoods of honey bees and impact their health and well-being. A1 said, “I think honey bees die more easily; the heat and the lack of water sometimes kill them, and they appear dead on the floor... Their lives will be shorter if they cannot feed and nourish themselves correctly”. A3 added that the hive population density is decreasing in his apiary, and climate change is the root cause because it affects the physiological development of honey bees and the queen’s reproductive capacity. Although farmers affirmed not keeping records of apiaries’ performance, they listed two common tendencies in hives’ health: (1) reduction of the honey bees’ lifespan; and (2) increase in honey bees’ mortality. A7 mentioned, “Just by seeing the hives, I quickly realized that honey bees suffer from the heat. I think the heat intensity makes them die or reproduce less quickly... I have had to join boxes [hives in different wooden boxes] because they have very few honey bees”.

Beekeepers more regularly attributed the increase in honey bee mortality to the physiological effects of climate change. Some beekeepers discussed how the everyday expressions of climate change have negatively impacted the integrity and safety of production environments and increased the apiaries’ vulnerability. For example, beekeepers reported flooding events that washed away entire apiaries. The increasingly common floods represent a limitation in the availability of space for placing apiaries. A1 said, “A couple of years ago, I had an apiary near the edges of the river, and when the storm came and lasted about four-to-five days, the river overflowed and washed away almost all the hives that were nearby... Some of the hives drowned, and others were completely carried away by the river… The hives that remained left because I could not go see them for several days... There was no access to where they were”. A4 added, “When the river overflows, the hives that are on the shore are always affected… The bees are always more stressed and sometimes even drown, so you have to look for places to put them, and the difficult thing is to find suitable places with water and food”.

With the increased frequency and intensity of the common climate change expressions, there has also been an increase in the incidence and intensity of pests and diseases. *Varroa destructor* is one of the pests that Salvadoran beekeepers have faced for many years. A2 said, “Varroa has been a problem for years... You can think it is controlled, but suddenly it is in the hives again... And it seems to be more aggressive”. The beekeepers perceived that this plague has strengthened in the last 10 years, and the aggressive nature of Varroa attacks puts the productivity of hives at risk and challenges beekeepers’ ability to manage and treat it. A4 pointed out the increased availability of products to treat Varroa, which has provided mediocre results, forcing beekeepers to take more drastic measures, such as sacrificing the hive. A5 said, “Varroa is getting stronger because we have not been able to control it even with the products we apply... It affects honey bees more in scorching seasons, perhaps because they are even more stressed and have higher work demands... Although we treat it, sometimes we have to dissolve the hive to treat it”.

With the arrival of the well-known Sahara dust, the beekeepers noticed the presence of new pests which, while not directly affecting the health of honey bees, does affect the quality of honey. Most of the beekeepers mentioned the yellow sugarcane aphid (*Melanaphis sacchari*). A7 explained how this pest feeds on the sap of sugarcane plants and attacks other crops, such as sorghum and corn. According to A7, this pest excretes a kind of molasses (produces a honeydew) that bees feed on and transform into honey. A6 added, “This pest eats sorghum, but when the sorghum in the area runs out, it advances to everything it finds… So, the problem is that the excretion remains on the plants, and when the bees are looking for food they find it, which is not good”. The reason this is not good is because the molasses that *M. sacchari* excretes become honey with specific organoleptic characteristics and chemical compositions that have less commercial potential. A7 said, “Since the yellow aphid appeared, well, look, I do not know much, but what they say is that the honey has a different flavor and aroma, and because of that, it costs more to sell the honey… People do not like it here and it is not good for exporters because it does not meet the requirements to sell in another country”.

The effects of climate change result in economic losses for beekeepers, who must work to keep hives healthy while addressing issues such as repairing damage to hive housing structures, providing water and feed, and managing pests and diseases. The following quotes allude to the economic challenges faced by Salvadoran beekeepers due to local climate variability: A1: “The flood damaged several boxes and wooden frames… Some of them broke. The ones that remained intact were already soaked with water, and that is sometimes bad because it attracts fungi that could be harmful to the bees and contaminate the honey... Buying all that again was super expensive, and I had to borrow [money] to pay for it”. A4: “Honey has dropped so much [in price] in recent years that sometimes I wonder if it is worth continuing to work on this... The bees no longer have water, and therefore it is difficult for them to produce more… I continue to invest labor and money in maintaining them and in the end, I am not able to recover what I have invested”. A7: “Pests and diseases are my headaches… Because if I do not control them on time, they damage my production and take the hive away. Luckily there are some products to control them; even though they are expensive, you cannot do it any other way”.

### 3.2. What Farm Management Practices Are Salvadoran Beekeepers Implementing as a Response Mechanism to Climate Change?

Due to local climatic variability, beekeepers have modified the traditional form of the physical structure (box and wooden frames) where they house honey bee hives. These modifications include using other materials that strengthen and protect honey bees from extreme climate conditions. The most commonly reported modification was using a combination of wood with metal applications on the upper and lower lids of the box to prevent fierce winds and rains from affecting the integrity of the hive. This modification also prevents the entry of pests that put honey bees’ health at risk. A5 mentioned, “With the lids [wood covered with a metal sheet], the box is kept more secure… The rain penetrates less into the hive and prevents the bees from drowning. The wind also enters less and therefore does not hurt the bees”. A3 added, “The wooden lids are there to protect the hive. Although I am still experimenting with them, I have heard they prevent the entry of some pests and predators... Although I have had them [wooden lids] for a shorth time, I know of others who have had them for several months and say that lids improve the hive’s health and that the rain affects them less”. Many highlighted the benefits of this modification, but others remained skeptical because they thought it could increase the hive’s internal temperature (A4, A7).

Another reported modification was placing pollen traps inside the wooden box. These traps are made of wood with a metal mesh designed to capture pollen and prevent the hive from becoming saturated. Beekeepers claimed that the traps help to avoid pollen saturation in the hive, but they are challenging to make and are not usually for sale. A4 explained, “With a large amount of pollen out there, we have had to implement pollen traps that help us manage the pollen in the hive and also harvest it for other uses”. Although the traps have proven effective in harvesting pollen, they pose a new problem because most beekeepers do not have the technical skills to process pollen, and ACCOPIDECHA needs particular technology to offer pollen management and processing services. A1 similarly stated, “Pollen traps have helped me control the overproduction there is, but then I have all that pollen that I do not know what to do with... I have seen that good medicinal products can be made that are natural, but I do not know how to do it… I do not know anyone using the pollen in the area”.

The beekeepers reported constantly relocating their apiaries to mitigate the impacts of climate change. First, they seek out places where extreme weather events (e.g., floods, winds, tropical storms) are not a threat. However, finding appropriate sites to place apiaries takes work because they must balance safety with food and water availability, which determines honey productivity. A7 said, “When looking for a piece of land to place an apiary, I try to ensure that the hives have at least a few trees around that give them shade, especially when it is too hot, and the sun is scorching… I am also interested in having clean water and flowers nearby or some source of food that the hives can use to collect nectar and pollen... It has been difficult for me lately because I look for places with no farmyard animals, and places with no houses or schools with people they can sting, and then I have problems”. A5 mentioned, “I am always looking to move my apiaries to places where they find more food and improve production… I have some apiaries far away, on land with no agricultural potential, which the owners let me use in exchange for a few jars of honey from every harvest”. Additionally, exposure to wind, sun, and rain, and proximity to water sources emerged as determining factors when choosing where to place apiaries and individual hives. A8 said, “With the rains and the winds, I always try to imagine if the apiaries would be at risk if something happened. Thank God nothing has happened to me, but you never know... You have to know how to place the apiaries in such a way that they can be protected and have everything they need”.

Due to the changes in apiary and hive locations, the practice of balancing the exposure of hives to light (sun) and shade has arisen. In the past, the light–shade ratio was 50–50; that is, 50% of the time, the hive was in direct sunlight, and the remaining 50%, the hive was out of the sun, entirely under shade. With changes in temperatures and rainfall, the beekeepers reported changing the percentages of light-shade to which they expose their apiaries. Although the 50–50 formula has historically been used, the beekeepers have experimented with new formulas, depending on where their hives are located and the local climate conditions. A9 said, “You have to balance the sun exposure that hives have because when it is hot, and they are directly exposed to the sun, they are in danger of suffocating… I try to balance the sun exposure between 70–30”. A3 added, “I do not expose the hives to too much sunlight because before, I did it to encourage productive activity. However, now I do not do it anymore; the heat affects them, and the sun only makes them worse—it makes them more aggressive and stresses them too much”. 

To respond to low food availability, beekeepers reported supplementing their bees’ food. Although beekeepers have yet to learn the science behind food supplementation for honey bees, their main intention was to relieve the pressure of finding food that climate change has placed on honey bees. The beekeepers used local food as sources of energy and protein. All of the beekeepers discussed preparing syrups as a source of energy, commonly using water, sugar, or honey, although using sugar was the most common. A4 said, “So that hives do not have such a hard time when the flora is scarce, I give them syrup that I prepare with water and dark sugar. Although it does not give them everything they need, it does help them be less stressed”. Honey was only used by beekeepers with leftovers from the previous harvest that they could not market due to low quality. A9 said, “The syrup is to give honey bees a source of energy to supplement the extra effort they use to collect nectar from the plants… I have almost always used water and dark sugar to prepare it, but last year when I had leftover honey from the harvest, I used it to prepare syrup for the hives... It would be better to use it than waste it... That honey was too dark”.

Beekeepers also used local food as sources of protein. Foods commonly used as a source of protein were seed and grain meals, including rice, squash, and morro. Due to the growing need to supplement food for honey bees, the commercialization and availability of chemical premade formulas has increased. However, beekeepers acknowledged high prices and said that they need to become more familiar with these products prior to using them. For example, A7 mentioned, “I have used several flours to supplement protein…. Nevertheless, sometimes preparing food with a seed meal is very complicated because you have to get the seed, prepare it, grind it, and then mix it with other ingredients... So, when you consider all of those steps, it is more feasible to buy a supplement... I bought one that they [other beekeepers] say is based on amino acids; it is called “Promoter L”… Although it is a little more expensive than the one I made, it can be found in any store that sells animal feed”.

### 3.3. What Are Salvadoran Beekeepers’ Needs and Preferences for Climate Change-Related Information as It Relates to Beekeeping?

Due to the increasing frequency and severity of climate change expressions, beekeepers constantly require more specific technical assistance to manage their apiaries and implement adaptability strategies. A1 commented, “Some apiary management practices are no longer efficient… For example, the light-shade relationship I used before is no longer the best, especially since it is much hotter, and the sun feels scorching… I have been experimenting with putting them in the sun for less time, but I do not know how to do it, and I would like to learn from someone who knows how to do it”. Most beekeepers emphasized their need for information and technical assistance to formulate supplementary diets and manage pests and diseases. A9 said, “I have discouraged myself many times from giving syrup or something else to the hives because I am afraid of preparing it and that instead of benefiting them, it might poison them or do them some harm… Although I see that others are doing it and that encourages me, I do not know if it is going to work for me”. A5 added, “It has not only been difficult for me with the diets... It has also been difficult to manage pests and diseases because I do not know much about useful chemicals... We need help implementing changes in apiary management that will ensure good honey... Having professional assistance for honey production would be an accomplished goal and a substantial benefit for all beekeepers”.

The beekeepers reported that their primary source of climate change information was from the internet. All of the beekeepers except for one accessed climate change information on the internet using their smartphone. Newspapers, television, and radio were not sources that the beekeepers used for information on the effects of climate change and adaptability. Although internet access continues to be a problem for beekeepers in El Salvador, it is even more challenging for them to understand the climate change information they find. The following quotes reflect the limitations that the beekeepers’ encountered regarding their ability to use and apply climate change information: A3: “I keep seeing things [information] about climate change on my cell phone… I have seen that it is becoming much stronger, and some things that I see in YouTube videos are similar to what happens to us here... However, how they are managing the lack of water or food would not work here, at least that is my perception”. A6: “I read very little, and I write with many difficulties... so it is of no use to me when they give me information that I cannot understand... I want someone to demonstrate how these things are done so I can replicate it in my apiary”. A9: “I know that climate change is a serious problem and that I must do something to manage it if I want to continue producing honey... my problem is, above all, that I do not know what information is relevant here... Although I know there is good information, I do not know what is applicable and what is not”.

Beekeepers’ difficulty in understanding climate change information is reduced when they access the information through interactions with other experienced beekeepers or an ACOPIDECHA technician. A3 said, “When A1 explained how to put the syrup in the hive, it became much easier for me, so much so that I already put the syrup in several times by myself. I have learned a lot by visiting other beekeepers’ apiaries because I go and do everything there with them”. Beekeepers preferred receiving technical assistance and training about climate change effects and adaptability from ACCOPIDECHA personnel and staff more than they preferred receiving technical assistance and training from other entities. A9 said, “They already know how apiaries are and work here, and they come and explain things to us in a super understandable way... They always show us how to manage the apiary... I prefer they come because we have a good relationship and I can ask them questions”. Regardless of which entity provided technical assistance, the beekeepers preferred personal interactions (one-on-one or group) with the facilitator, specialized agent, or other beekeepers when receiving information about climate change effects and adaptation. A5 and A9 discussed the learning benefits that resulted from one-on-one interactions, including the ability to see certain practices demonstrated in the field and ask questions or resolve concerns in a safe space.

Although beekeepers had access to different sources of information on climate change adaptability, they trusted the information they received from ACCOPIDECHA. A9 said, “I am very interested in learning how to be more productive and to take better care of my hives... I have already learned a lot since I joined the association... I trust what they are teaching me because I already see that it works. I notice their interest in making every associate better... They want us to improve our honey production because, in the end, the association improves”.

## 4. Discussion

Climate change has direct and indirect implications on the physiological development and daily performance of all animal and plant species worldwide [56,57]. Bees are currently among the species of most significant interest for protection and conservation. This is mainly due to the impacts of climate change on the productivity potential of wild beehives that produce honey and perform pollination, as well as solitary wild bee species that perform pollination [20,21,56]. Climate change has drastically reduced the habitat and resources available to bees in El Salvador [46]. Findings from the current study regarding Salvadoran beekeepers’ awareness about climate change impacts are consistent with those from previous studies that have reported that Salvadoran beekeepers noticed reductions in the availability of local flora and changes in flowering times [46,58]. Similar findings have also been documented regarding beekeepers’ perceptions about reduced water sources and drought increases. These perceptions align with national meteorological information reports claiming an 800% increase in the incidence of extreme weather events and droughts every decade since 1980 [42]. Climate change has reduced Salvadoran surface aquifers by an average of 13 feet and contributed to increasing the current 90% of surface water pollution [42].

Although this study only explored the perceived impact of climate change on managed honey bees and not wild honey bees, there is literature reporting similar consequences to both populations globally [15,59]. Findings from the current study regarding beekeepers’ perceptions of how climate change affects honey bee longevity and well-being support those reported in [60], who stated that honey bee longevity has halved in the last 50 years. Ref. [60] also experimented with honey bees in captivity and recorded an average lifespan of 17.7 days, which is less than the 34.3-day average lifespan reported in the 1970s. Based on their results, ref. [60] projected losses of up to 33% of honey bee populations annually. The increased physiological demands that climate change places on honey bees (e.g., greater need for water), along with other climate change implications, are responsible for this increase in honey bee mortality [34,60,61,62]. For example, floods have caused losses in apiaries and honey production for Salvadoran beekeepers. According to a report from the Inter-American Development Bank, between 1990 and 2012, there were 2100 flood events caused by climate change [63]. Although economic losses vary for each event, it is common for agriculture to be one of the economic sectors reporting losses in the millions [46,64].

Climate change has also increased the resistance and aggressiveness of pests and diseases associated with honey bees—a finding that has been widely reported [23,31,65,66]. *V. destructor* is one of the most significant pests to beekeepers worldwide [66,67,68]. This pest reduces beehive life expectancy drastically, which is why it tops the list of reasons for economic losses associated with beekeeping [46,66,67,68,69,70,71]. Salvadoran beekeepers affirmed an increase in the incidence of *V. destructor* due to climate change, which is in accordance with national reports of increased *V. destructor* activity due to rising temperatures and humidity [71]. Another species of growing interest in beekeeping and other Salvadoran agricultural sectors is the yellow sugarcane aphid, whose presence is attributed to the arrival of dust from the Sahara. According to the El Salvador Environmental Observatory, dust from the Sahara consistently reaches El Salvador, causing changes in humidity and temperature. As a result, pests and organisms that are not endemic to the country arrive [72]. The yellow sugarcane aphid reduces honey quality by adding sugars from C4 plants, while honey bees typically rely on C3 plants as their primary food source [73,74]. The Codex Alimentarius establishes the parameters and regulations for food production and commercialization and recognizes C4-derived honey as suitable food for human consumption [75]. However, the presence of C-4-derived honey and other changes in organoleptic properties could limit its commercialization potential [73]. When exporting honey, Salvadoran beekeepers find greater acceptance and market opportunities for clear honey. Dark honey and other flavored honey have opportunities in niche markets, but those opportunities are scarcer and sometimes come with less bargaining power for the beekeepers [46,73]. Providing consumers with information about the higher nutraceutical qualities of honeydew honey (e.g., greater antioxidant power, greater content of mineral elements, greater antibacterial effects) could increase their perceived value of the product and, as a result, increase its market potential.

To adapt to climate change, the interviewed Salvadoran beekeepers have implemented changes in their farm management practices, all of which have increased their labor requirements and management costs [23]. For example, modifying the traditional hive housing structure is a common new farm management practice. However, this practice is still under experimentation, and many of its effects are unknown, mainly due to a lack of records indicating its performance. The use of pollen traps is also a relatively new and common practice that Salvadoran beekeepers are implementing. This practice stems from the high volumes of pollen harvested by bees, which often exceeds the capacity of the hive and accumulates, hindering the bees’ labor. The increase in the amount of pollen in local flora reported by Salvadoran beekeepers in the current study contradicts the results reported by [24], who found an opposite trend in the availability of pollen in local flora in Piedmont, Italy. There are several possible explanations for this discrepancy. One is that the local flora are different between the two geographic areas and may respond differently to changes in climate [76,77,78]. Another is that climate change expressions in Europe are different and relatively milder than those in America, especially in Central America [79,80].

On average, Salvadoran temperatures have increased by 1.3 °C since 1950, and Chalatenango is among the states with the most significant temperature increase [42]. This temperature increase has prompted beekeepers in the area to reconsider the historical formula regarding the relationship between light (50%) and shade (50%) to which their hives are exposed. [81] reported an internal hive temperature of 95 °F as ideal for maintaining the eggs, larvae, and pupae of honey bee hives. However, in Chalatenango, temperatures of up to nearly 109 °F have been recorded. High temperatures and direct exposure to sunlight can rapidly increase a hive’s internal temperature. In this case, the bees must work harder to regulate the internal temperature, resulting in behavioral deficits [82]. However, the literature also suggests that managed bees can perform adequately in permanent exposure to the sun [83]. Nevertheless, this practice must be evaluated by considering the apiary’s local temperature conditions, location, and the availability of food and water sources [84,85]. Choosing the apiary location is a challenge for Salvadoran beekeepers because there are no clear guidelines for optimal beekeeping practices [86]. Although several national and international entities have developed sets of practical beekeeping guidelines, there is a lack of specific regulations regarding suitable apiary locations [87,88]. With both agricultural and forest lands decreasing in El Salvador, the availability of suitable apiary locations is a growing challenge for beekeepers [89].

Food supplementation for honey bees is widely studied by entomologists and beekeepers [90,91]. Similar to Salvadoran beekeepers, beekeepers in other parts of the world are providing additional food sources to managed honey bees as a climate change adaptation strategy [23,24,91,92]. Recent studies have demonstrated how supplementing food has positive effects on larval development and improves the labor potential of worker bees [93,94]. The practice of food supplementation for honey bees occurs mainly in the dry seasons (November–May) in El Salvador. However, in other countries (e.g., United States, Italy), honey bee food is only supplemented during the periods of greatest need and stress for the hives [24,94]. For a long time, honey bee diets were mainly artisanal or homemade, but recently, there has been an increase in the availability and variety of pre-manufactured food supplements [95]. Although information is available regarding the pros and cons of supplementing food for honey bees, there are still gaps in the literature, primarily related to the variation in the hives’ nutritional requirements throughout the year [94].

Salvadoran beekeepers are changing their apiary management practices to adapt to climate change, but they feel unprepared to deal with this complex environmental problem. Beekeepers have a constant need for technical assistance to cope with climate change [96]. This study’s findings are consistent with those of other studies that have demonstrated the inefficiencies of technical assistance and agricultural extension services in the developing world [97]. In many countries, third parties (e.g., non-profit organizations, private companies) complement the agricultural extension services offered by the central or local government [98,99]. However, these efforts still fall short of meeting the needs of farming communities [34,38,98,99]. Agricultural producers in low- and middle-income countries, including beekeepers in El Salvador, require more technical assistance to implement effective and efficient climate change adaptability strategies [34,99,100]. One of the main challenges for the Salvadoran beekeepers interviewed was their difficulty in understanding available climate change-related information about beekeeping. Other studies have similarly highlighted how the lack of relevant information limits agricultural producers’ ability to implement climate change adaptation strategies [100]. The variation in language used to discuss climate change and adaptation strategies also makes it difficult for those who need the information (e.g., agricultural producers) to understand and use the information [101,102].

## 5. Conclusions

Findings from the current study can be generalized to beekeepers in Chalatenango, El Salvador, who are also ACCOPIDECHA members, but not beyond this particular region or association. Nevertheless, there may be similarities in the climate change experiences of beekeepers living in similar geographic regions in developing countries. Overall, beekeepers in El Salvador noticed climate change-related challenges and expressions (e.g., water and food scarcity, increasing temperatures, extreme weather-related events such as flooding) that negatively affected the health, safety, and productivity of honey bees, consequently impacting the beekeepers’ livelihoods. To adapt to these challenges, beekeepers implemented various strategies, such as modifying the physical structure (box and wooden frames) where honey bee hives were housed, placing pollen traps inside the box, relocating apiaries, changing the light–shade ratio to which the hives were exposed, and supplementing the honey bees’ food. The beekeepers expressed a need for information and technical assistance in implementing climate change adaptation strategies, especially in supplementing honey bee diets and managing pests and diseases. Because beekeepers found it difficult to understand climate change-related information about beekeeping that they received from the internet, they preferred receiving such information through personal interactions (one-on-one or group) with trusted ACCOPIDECHA personnel and staff. Further research is needed to investigate the long-term effects of some climate change adaption strategies in El Salvador (e.g., modified hive structure), as their implications for honey bee health and well-being are largely unknown. There is also a need for research investigating effective communication approaches, strategies, and channels to help Salvadoran beekeepers access and make use of climate change-related information about beekeeping. It is imperative that Salvadoran beekeepers receive information and demonstrations to improve their climate change adaptation strategies and implement new ones to address the challenges they experience. Finally, the findings of this study highlighted the urgent need for bee conservation and beekeeping policies in El Salvador, such as establishing a payment scheme to compensate managed bee producers for the environmental services provided by their honey bees.

## Data Availability

The data are available from the corresponding author upon request.

## Supplementary Materials

The following supporting information can be downloaded at: https://www.mdpi.com/article/10.3390/insects14060493/s1, File S1: Interview Protocol - Honey Production and Climate Change: Beekeepers’ Perceptions, Farm Adaptation Strategies, and Information Needs.
