# Supplementary material for: Honey Production and Climate Change: Beekeepers’ Perceptions, Farm Adaptation Strategies, and Information Needs
Source: Insects. 2023 May 25;14(6):493. doi: 10.3390/insects14060493 (PMC10299425; doi:10.3390/insects14060493)
Supplement: Supplementary file 1 [file insects-14-00493-s001.zip › insects-2336894-supplementary.pdf]

Interview Protocol - Honey Production and Climate Change: Beekeepers' Perceptions, Farm Adaptation Strategies, and Information Needs

1. How has the climate changed locally in the last ten years?
2. How is climate change affecting local ecosystems, including those where you have an apiary?
3. How would you describe apiaries' behavioral changes in the last ten years (reproduction, production, life-span)?
4. What do you think are the reasons for the reduction of bee communities in the ecosystems in which you work?
5. How is climate change influencing how you manage your apiary(ies)?
6. How prepared do you think you are to cope with climate change in your production?
7. What are the resources you have available to assist you with climate adaptability processes (credits, technical assistance, information)?
8. Who is supporting you to cope with the current climate scenarios (local or central government, NGOs, academics)?
9. What climate information needs are you experiencing? Where are you looking for that information? How easy is it for you to access?
10. How is climate change changing the quality of your honey production?
11. How do local/national markets respond to your current honey production?
12. What are your training needs to cope with climate change effectively?
